# Effect of host breeds on gut microbiome and serum metabolome in meat rabbits

**DOI:** 10.1186/s12917-020-02732-6

**Published:** 2021-01-07

**Authors:** Xiaoxing Ye, Liwen Zhou, Yao Zhang, Shuaishuai Xue, Qian Fu Gan, Shaoming Fang

**Affiliations:** College of Animal Science (College of Bee Science), Fujian Agriculture and Forestry University, Fuzhou, China

**Keywords:** Host breeds, Gut microbiome, Serum metabolome, Meat rabbits

## Abstract

**Background:**

Gut microbial compositional and functional variation can affect health and production performance of farm animals. Analysing metabolites in biological samples provides information on the basic mechanisms that affect the well-being and production traits in farm animals. However, the extent to which host breeds affect the gut microbiome and serum metabolome in meat rabbits is still unknown. In this study, the differences in phylogenetic composition and functional capacities of gut microbiota in two commercial rabbit breeds Elco and Ira were determined by 16S rRNA gene and metagenomic sequencing. The alternations in serum metabolome in the two rabbit breeds were detected using ultra-performance liquid chromatography system coupled with quadrupole time of flight mass spectrometry (UPLC-QTOFMS).

**Results:**

Sequencing results revealed that there were significant differences in the gut microbiota of the two breeds studied, suggesting that host breeds affect structure and diversity of gut microbiota. Numerous breed-associated microorganisms were identified at different taxonomic levels and most microbial taxa belonged to the families Lachnospiraceae and Ruminococcaceae. In particular, several short-chain fatty acids (SCFAs) producing species including *Coprococcus comes*, *Ruminococcus faecis*, *Ruminococcus callidus*, and *Lachnospiraceae bacterium NK4A136* could be considered as biomarkers for improving the health and production performance in meat rabbits. Additionally, gut microbial functional capacities related to bacterial chemotaxis, ABC transporters, and metabolism of different carbohydrates, amino acids, and lipids varied greatly between rabbit breeds. Several fatty acids, amino acids, and organic acids in the serum were identified as breed-associated, where certain metabolites could be regarded as biomarkers correlated with the well-being and production traits of meat rabbits. Correlation analysis between breed-associated microbial species and serum metabolites revealed significant co-variations, indicating the existence of cross-talk among host-gut microbiome-serum metabolome.

**Conclusions:**

Our study provides insight into how gut microbiome and serum metabolome of meat rabbits are affected by host breeds and uncovers potential biomarkers important for breed improvement of meat rabbits.

**Supplementary Information:**

The online version contains supplementary material available at 10.1186/s12917-020-02732-6.

## Background

Gut microbial communities play pivotal roles in host nutrient digestion, energy harvesting, immunity modulation, and disease development [1]. Recently, compositional and functional variation in the gut microbiota has been linked to the health and production performance of farm animals [2]. Thus, understanding the mechanisms governing the maintenance and function of gut microbiota is crucial for farm animal industry. Both environmental (e.g., diet, medicine, and environmental hygiene) and host factors (e.g., genetics background, gender, and age) can shape the gut microbial communities [3]. In recent years, accumulating evidence has highlighted the variation of gut microbiome in different animal breeds fed under the same conditions. For example, Xiao et al. found that gut microbiota of Landrace and Yorkshire pigs were similar but remarkably different from that of Duroc and Hampshire pigs [4]. Pandit et al. identified several breed-specific biomarkers including the genera *Clostridium*, *Blautia*, *Butyrivibrio*, *Ruminococcus*, and *Roseburia* in the gut microbial communities of different broiler chicken breeds [5], while Cheng et al. observed substantial changes in the metabolic capacities of xylose, ribose, and fucose in the gut microbiome of Lantang and Duroc pigs [6].

Metabolomics is an omics approach to identify and quantify all metabolites present in biological samples [7]. Characterizing the metabolic profile of an individual can comprehensively reflect the final consequences of complex biological interactions of genetic and environmental factors [8]. In addition, circulating metabolites constitute the basic biological mechanisms that affect the well-being and production traits in farm animals [9]. Previous studies have unraveled a variety of breed-associated metabolic molecules in different animals raised under the same environmental conditions. For instance, Italian Large White pigs were distinguished from Italian Duroc pigs by plasma levels of sphingomyelins and biogenic amine [9]; several serum fatty acids (e.g., oleic acid and linoleic acid), amino acids (e.g., glutamine and asparagine), and organic acids (e.g., citric acid and fumaric acid) were regarded as breed-specific biomarkers in different beef cattle breeds [8]; and certain amino acids and organic acids in both muscle and liver have been shown to reflect the breed-selection history of Dorper and Merino sheep [10].

Microbiomics and metabolomics have been effective in improving our understanding of how host breeds affect gut microbiome and metabolic profile in livestock, respectively. Nonetheless, few studies have applied a combined meta-omics approach to evaluate the role of host breeds in structuring gut microbial communities and circulating metabolic profiles, and to investigate the relationship between breed-associated microorganisms and metabolites. In the current study, we analysed the gut microbiome and serum metabolome in two commercial meat rabbit breeds (Elco and Ira), exploring the correlations between breed-associated microbial species and serum metabolites. Our findings not only provide the basic knowledge of how host breeds shape both the gut microbiome and the serum metabolome, but uncover potential biomarkers for practical applications in promoting the well-being status and production performance of meat rabbits.

## Results

### Structure and diversity of gut microbiota in Elco and Ira rabbits

**Fig. 1:**
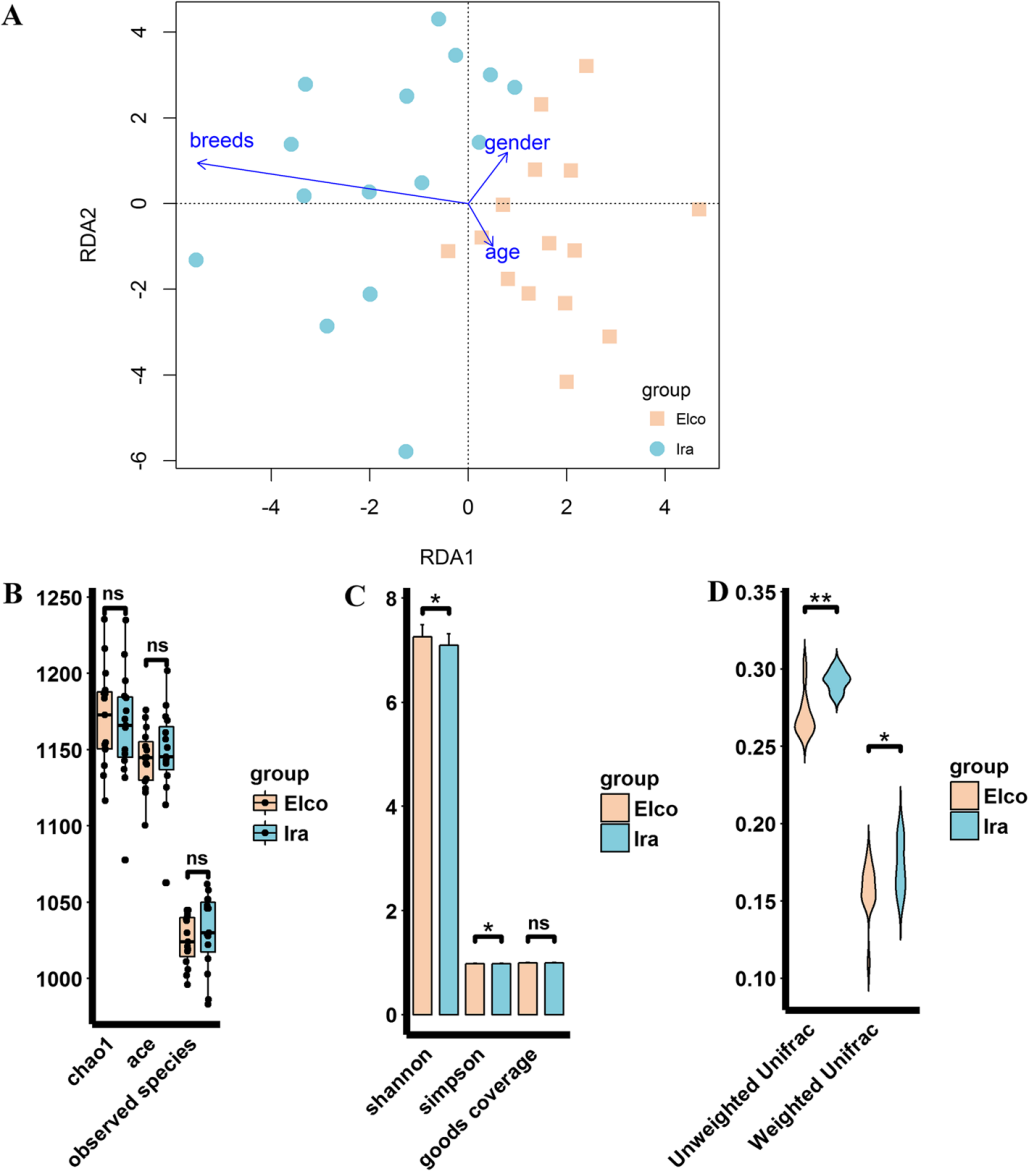
Differences in structure and diversity of gut microbiota between Elco and Ira rabbits. **a** RDA analysis exhibited the effect of host breeds, gender and age on gut microbial community structure. **b** The differences in Chao1, ACE, and observed species indices between Eloc and Ira rabbits (“ns” FDR adjusted *p* > 0.05). **c** The differences in Shannon, Simpson, and Good’s coverage indices between Eloc and Ira rabbits (“*” FDR adjusted *p* < 0.05). **d** The differences in unweighted and weighted UniFrac distance between Eloc and Ira rabbits (“**” FDR adjusted *p* < 0.01)

To understand how host breeds affect gut microbial community structure, we performed RDA analysis, which revealed that breeds exerted a stronger effect on gut microbial communities in comparison to gender and age (Fig. 1a). The alpha diversity analysis showed that there were no significant differences in Chao1, ACE, observed species, and Good’s coverage between Elco and Ira rabbits, but Elco rabbits had significantly higher Shannon and Simpson indices than Ira rabbits (Fig. 1b and c, FDR adjusted *P* < 0.05). On the other hand, beta diversity analysis using both weighted and unweighted UniFrac distances indicated that Ira rabbits had higher dissimilarities among gut microbial communities than Elco rabbits (Fig. 1d, FDR adjusted P < 0.05).

### Differences in gut microbial composition between Elco and Ira rabbits

**Fig. 2:**
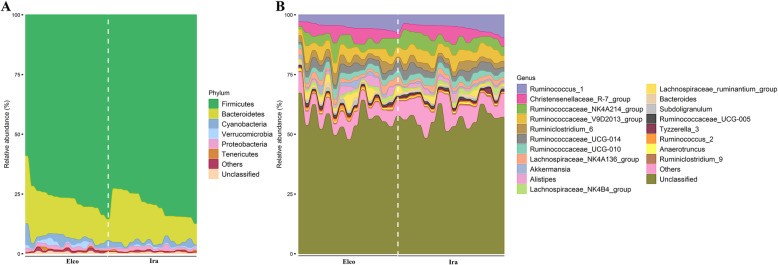
The gut microbial taxonomic distribution in Elco and Ira rabbits. (**a**) At the phylum level. **b** At the genus level

The six predominant phyla in the gut microbial communities of both Elco and Ira rabbits were as follows: Firmicutes, Bacteroidetes, Cyanobacteria, Verrucomicrobia, Proteobacteria, and Tenericutes (Fig. 2a). Sixteen out of nineteen most dominant genera were derived from the phylum Firmicutes, such as *Ruminococcus_1*, *Christensenellaceae_R-7_group*, *Ruminococcaceae_NK4A214_group*, and *Ruminococcaceae_V9D2013_group* (Fig. 2b). The other three most dominant genera were *Alistipes* and *Bacteroides* (phylum Bacteroidetes) and *Akkermansia* (phylum Verrucomicrobia).

Wilcoxon rank sum test analysis was performed to identify differences in relative abundances of bacteria between Elco and Ira rabbits within the two taxonomic levels: phylum and genus (Additional file 2: Table S2). At the phylum level, the relative abundance of Verrucomicrobia in Elco rabbits (1.21±0.93) was significantly higher in comparison to Ira rabbits (0.50±0.28; FDR adjusted *P* < 0.05), but there were no significant differences in relative abundances of the other phyla. At the genus level, the relative abundances of *Akkermansia*, *Lachnospiraceae_ruminantium_group*, *Subdoligranulum*, and *Ruminococcus_2* were significantly higher in Elco rabbits (1.18±0.94, 2.08±1.98, 0.81±0.48, and 0.73±0.40, respectively) compared to those of Ira rabbits (0.50±0.28, 0.78±0.86, 0.47±0.23, and 0.36±0.15, respectively;FDR adjusted P < 0.05), while the relative abundances of other genera did not differ significantly (Additional file 2: Table S2).

**Fig. 3:**
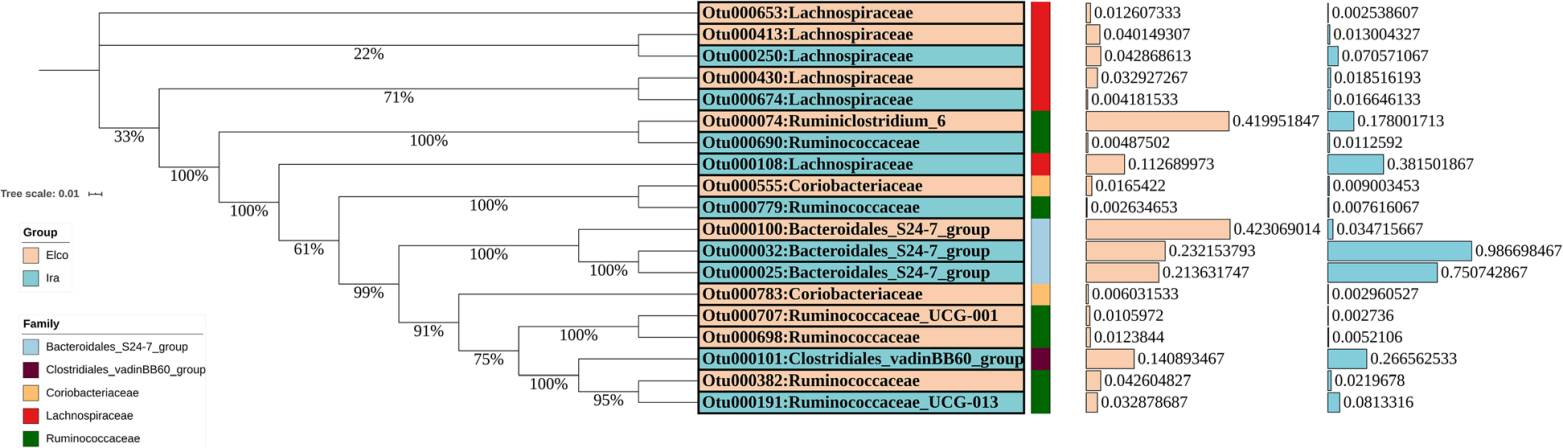
The identified significantly different OTUs in Elco and Ira rabbits. The neighbor-joining tree represents phylogenetic relationships of the breed-associated OTUs and bootstrap values are shown on the branches. The barplot shows the average relative abundances of each OTU in the two rabbit breeds

To detect more differentially enriched bacteria between rabbit breeds, we analysed the relative abundances of OTUs using the Wilcoxon rank sum test. Nineteen OTUs exhibited significantly different abundances between Elco and Ira rabbits (Fig. 3 and Additional file 2: Table S3). Among these, ten OTUs were enriched in Elco rabbits and nine other OTUs were augmented in Ira rabbits. These OTUs were annotated to different taxonomic levels. In Elco rabbits, three OTUs were annotated to the family Lachnospiraceae, two OTUs to each of the family Ruminococcaceae and Coriobacteriaceae, and one OTU to the family Bacteroidales_S24-7_group. At the genus level, one OTU was annotated to each of *Ruminiclostridium_6* and *Ruminococcaceae_UCG-001*. In Ira rabbits, eight OTUs were annotated to the family level, including three OTUs to Lachnospiraceae, two OTUs to each of Ruminococcaceae and Bacteroidales_S24-7_group, and one OTU to Clostridiales_vadinBB60_group. One OTU was annotated to the genus *Ruminococcaceae_UCG-013*.

**Fig. 4:**
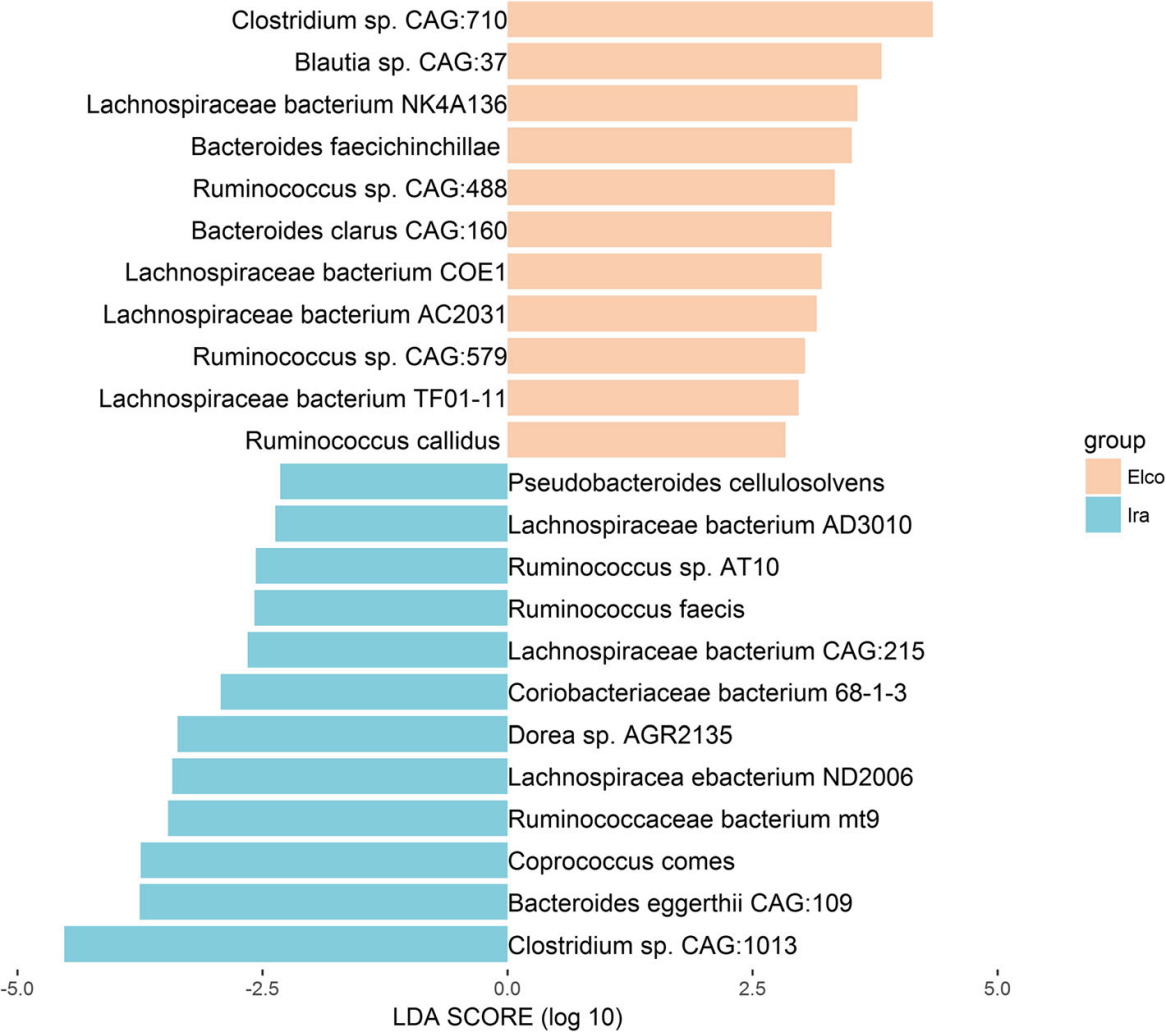
The identified significantly different microbial species in Elco and Ira rabbits. The apricot bar represents for Elco-associated species, the cyan bar corresponds to Ira-associated species

Due to the limitations of 16S rRNA gene sequencing, we could not identify the microorganisms at the species level. Hence, we performed LEfSe analysis using metagenomic species data. As shown in Figs. 4, 11 species, including *Clostridium sp. CAG:710*, *Blautia sp. CAG:37*, *Lachnospiraceae bacterium NK4A136*, *Bacteroides faecichinchillae*, and *Ruminococcus sp. CAG:488* were more abundant in Elco rabbits, while 12 species, including *Clostridium sp. CAG:1013*, *Bacteroides eggerthii CAG:109*, *Coprococcus comes*, *Ruminococcaceae bacterium mt9*, and *Lachnospiracea ebacterium ND2006* were more abundant in Ira rabbits. In consistent with the results obtained at higher taxonomic levels, we found that in both rabbit breeds most of the more abundant species (16 out of 23) were members of the family Lachnospiraceae and Ruminococcaceae.

### Microbial functional profiles in Elco and Ira rabbits

**Fig. 5:**
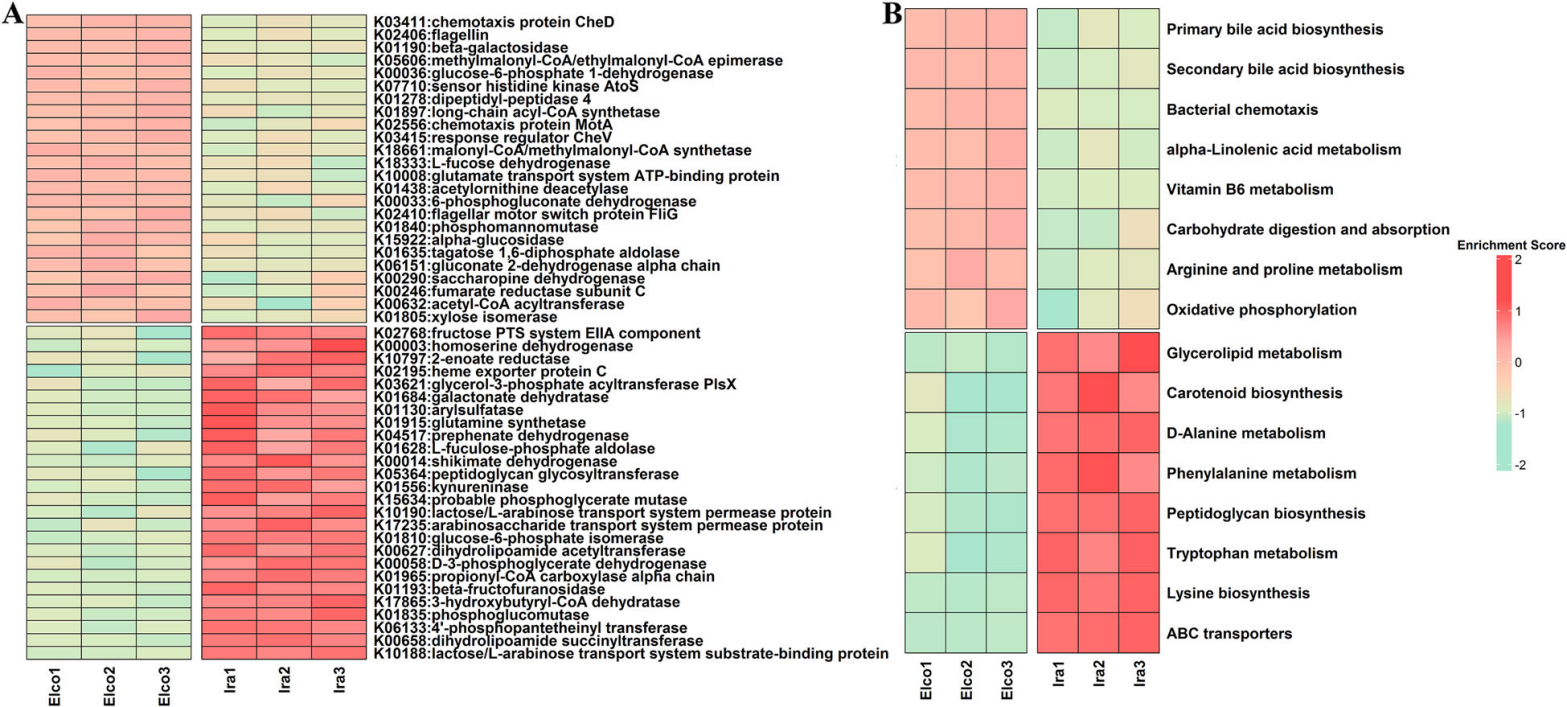
Heatmap showing differentially enriched functional capacities in Elco and Ira rabbits. **a** KOs. **b** KEGG pathways

To investigate the functional differences of the gut microbial communities between Elco and Ira rabbits, LEfSe analysis was performed using metagenomic KOs and KEGG pathways data (Fig. 5 and Additional file 2: Table S4). Twenty-four KOs were highly represented in Elco rabbits (Fig. 5a), of which most were assigned to bacterial chemotaxis (e.g., K03411, K02556, and K02410), two-component system (e.g., K02406, K07710, and K03415), pentose phosphate pathway (e.g., K00036, K00033, and K06151), fructose and mannose metabolism (e.g., K18333, K01840, and K01805), and valine, leucine and isoleucine degradation (e.g., K05606 and K18661). Meanwhile, 26 KOs were significantly enriched in Ira rabbits, most of which were related to ABC transporters system (e.g., K10188, K17235, K10190, and K02195), phenylalanine, tyrosine and tryptophan metabolism (e.g., K01556, K00014, K04517, and K10797), galactose metabolism (e.g., K01835, K01193, and K01684), glycerolipid metabolism (e.g., K01130 and K03621), and lysine metabolism (e.g., K00658 and K00003). KEGG pathways comparison analysis indicated that eight functional categories, including primary bile acid biosynthesis, secondary bile acid biosynthesis, bacterial chemotaxis, and alpha-linolenic acid metabolism, were more active in Elco rabbits. In contrast, 8 functional categories, such as, ABC transporters, lysine biosynthesis, tryptophan metabolism, peptidoglycan biosynthesis, and phenylalanine metabolism, were more abundant in Ira rabbits (Fig. 5b).

### Serum metabolome alterations in Elco and Ira rabbits

**Fig. 6:**
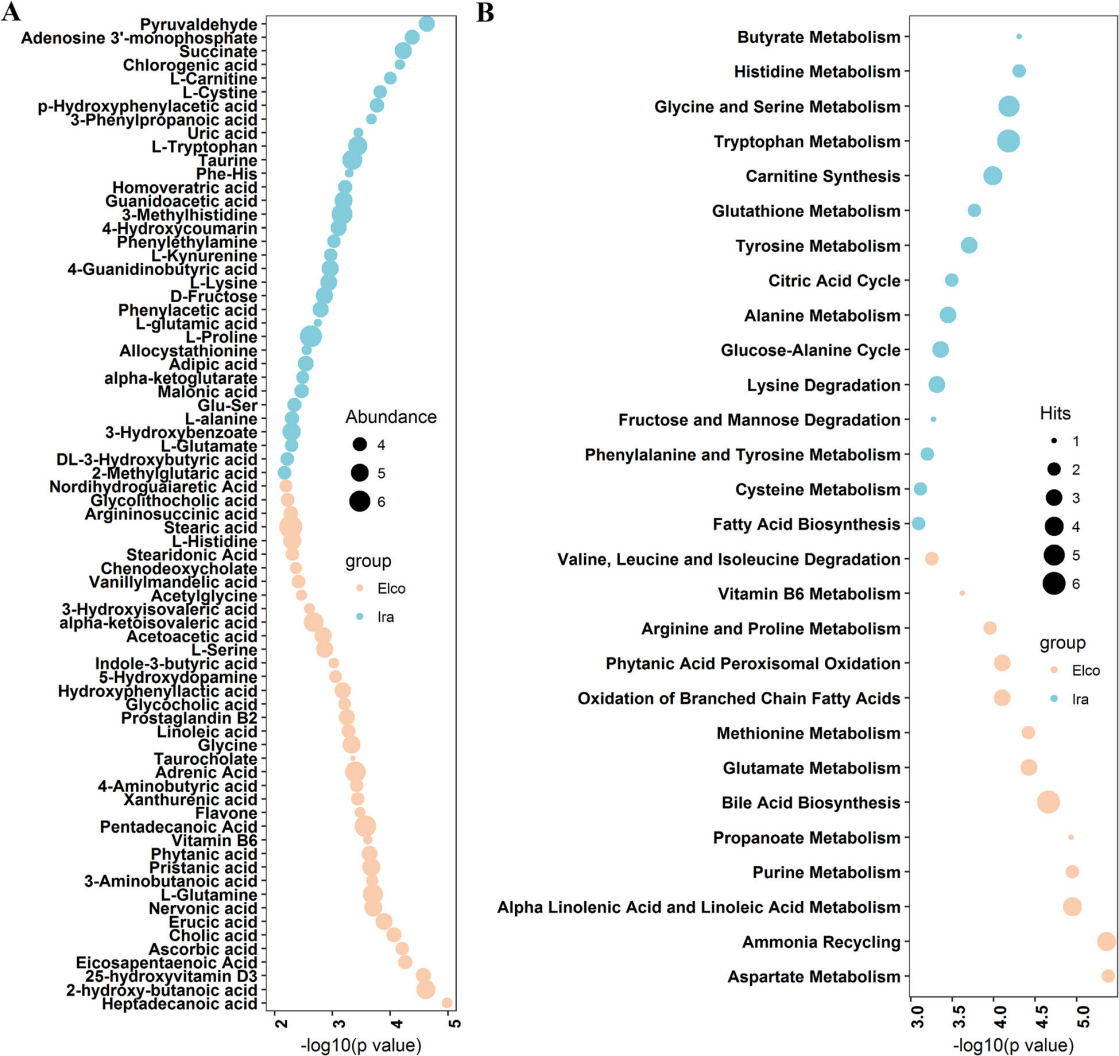
The differences in serum metabolic profiles of Elco and Ira rabbit. **a** Significantly different metabolites. **b** KEGG pathways enriched by significantly different metabolites

To comprehensively understand how the serum metabolome differed between Elco and Ira rabbits, non-targeted UPLC-QTOFMS was used to characterise the serum metabolomics profiles. In total, 834 metabolite features were obtained. PLS-DA analysis using all metabolite features showed a clear difference between Elco and Ira rabbits (Additional file 1: Fig. S1). In addition, 73 significantly different metabolites were identified (FDR adjusted *p* < 0.05, Fig. 6a, Additional file 2: Table S5). Among these, 39 metabolites were more abundant in Elco rabbits, with 14 fatty acids and derivatives (e.g., heptadecanoic acid, erucic acid, nervonic acid, pristanic acid, phytanic acid, and pentadecanoic acid); six amino acids and derivatives (e.g., L-glutamine, glycine, L-serine, and acetylglycine); and six organic acids (e.g., 2-hydroxy-butanoic acid, 3-aminobutyric acid, glycolithocholic acid, and 4-aminobutyric acid). The other 34 metabolites were enriched in Ira rabbits, including 13 amino acids and derivatives (e.g., L-cystine, 3-methylhistidine, L-kynurenine, L-lysine, L-proline, and L-tryptophan), ten organic acids (e.g., chlorogenic acid, 3-phenylpropanoic acid, phenylacetic acid, succinate, and alpha-ketoglutarate), and two fatty acids and derivatives (2-methylglutaric acid and adipic acid).

Pathway enrichment analysis using significantly different metabolites identified 28 differentially enriched metabolic pathways in the two rabbit breeds (Fig. 6b). Several metabolic pathways of fatty acids (e.g., alpha linolenic acid and linoleic acid metabolism, propanoate metabolism, bile acid biosynthesis, and oxidation of branched chain fatty acids) and amino acids (e.g., aspartate metabolism, glutamate metabolism, arginine and proline metabolism, and valine, leucine and isoleucine degradation) were more activated in Elco rabbits, while in Ira rabbits amino acids metabolic pathways (e.g., histidine metabolism, glycine and serine metabolism, tryptophan metabolism, glutathione metabolism, and tyrosine metabolism) were more activated.

### Correlations between breed-associated gut microbial species and serum metabolites

**Fig. 7:**
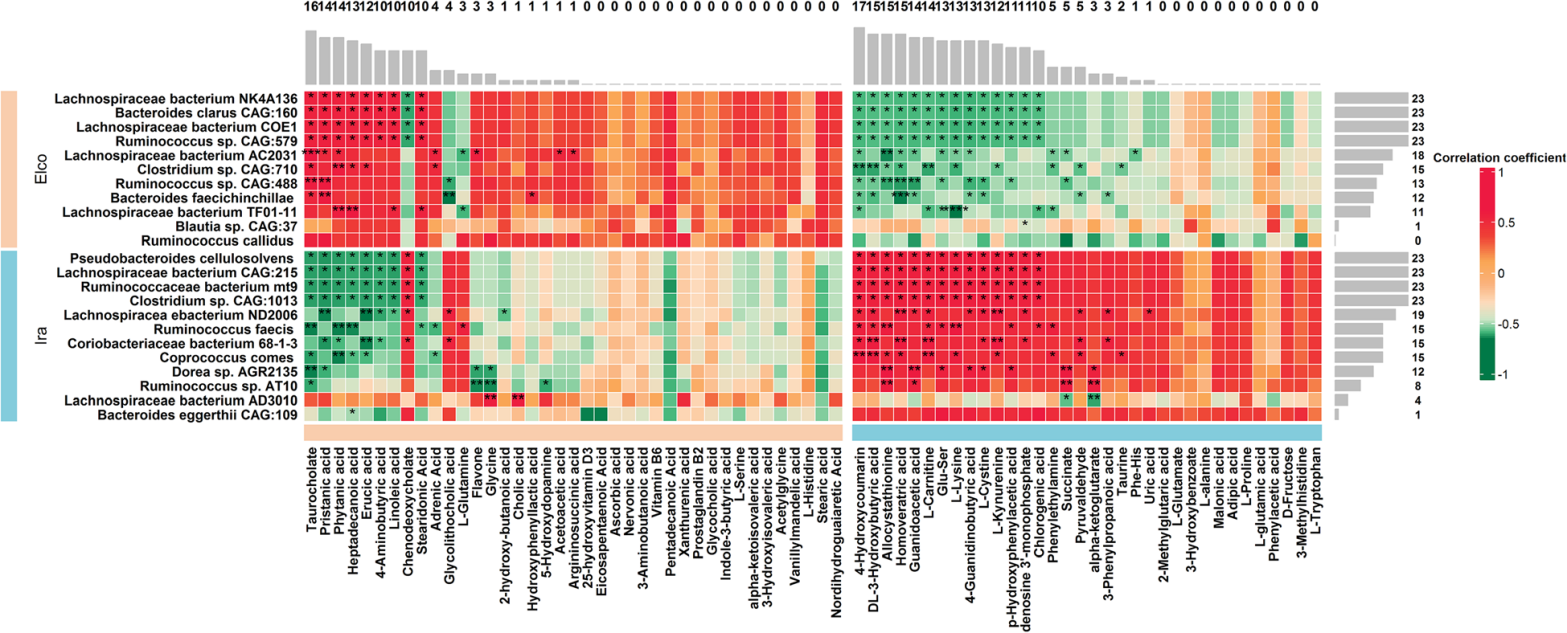
Heatmap showing correlations between breed-associated microbial species and serum metabolites. The barplot no the top and right side of heatmap shows the total amount of significant correlations of each breed-associated metabolites and species, respectively. “*” FDR adjusted p < 0.05, “**” FDR adjusted p < 0.01, “***” FDR adjusted *p* < 0.001, “****” FDR adjusted *p* < 0.0001

To evaluate the potential relationships between breed-associated gut microbial species and serum metabolites, we performed Spearman rank correlation analysis. A total of 343 significant correlations were identified (FDR adjusted *P* < 0.05, Fig. 7). In Elco rabbits, ten of the most abundant species were significantly associated with at least one metabolite. Among these, *Lachnospiraceae bacterium NK4A136*, *Bacteroides clarus CAG:160*, *Lachnospiraceae bacterium COE*1, *Ruminococcus sp. CAG:579*, and *Lachnospiraceae bacterium AC2031* were the predominant species, which had 110 significant associations with metabolites. Meanwhile, twenty Elco-associated metabolites showed significant associations with at least one species. Among the metabolites, taurocholate, pristanic acid, phytanic acid, heptadecanoic acid, erucic acid, 4-aminobutyric acid, linoleic acid, chenodeoxycholate, and stearidonic acid were the dominant ones, each of which was associated with at least 10 species. Twelve Ira-associated species, including *Pseudobacteroides cellulosolvens*, *Lachnospiraceae bacterium CAG:215*, *Ruminococcaceae bacterium mt9*, *Clostridium sp. CAG:1013*, and *Lachnospiracea ebacterium ND2006*, which totally exhibited 181 significant associations with 35 metabolites. In addition, 22 Ira-associated metabolites were significantly correlated with at least one species, including 4-hydroxycoumarin, DL-3-hydroxybutyric acid, allocystathionine, homoveratric acid, guanidoacetic acid, L-carnitine, Glu-Ser, L-lysine, 4-guanidinobutyric acid, L-cystine, L-kynurenine, p-Hydroxyphenylacetic acid, and adenosine 3′-monophosphate, each of which was associated with more than 10 species.

## Discussion

Emerging evidence has linked the gut microbiome and metabolome to the health, development, and growth of farm animals [11–13]. Hence, understanding how host and environmental factors affect the gut microbiome and metabolome is beneficial to improve the well-being and production performance of animals. However, few studies have investigated the effects of host breed on the gut microbiome and metabolome of meat rabbits. Thus, we explored differences in the gut microbiome and serum metabolome of Elco and Ira rabbits and established correlations between breed-associated microbial species and serum metabolites.

We evaluated whether breed factors could alter the gut microbial community structure, and similar to previously findings [14], we found that host breeds exerted a greater effect on the structure of gut microbiota than gender and age (Fig. 1A). The weak effect of gender and age on gut microbial communities is most likely due to the rabbit population used in this study, where animals were sexually immature individuals of a similar age [15, 16]. The Shannon and Simpson indices of Elco rabbits were significantly higher than those of Ira rabbits, whereas Ira rabbits showed significantly greater weighted and unweighted UniFrac distances than Elco rabbits (Fig. 1b-d). Earlier studies have highlighted these microbial diversity indices vary greatly in different pig, chicken, and horse breeds [4, 5, 17].

Consistent with previous studies on gut microbiota in meat rabbits [18, 19], we found that phyla Firmicutes, Bacteroidetes, Cyanobacteria, Verrucomicrobia, Proteobacteria, and Tenericutes, and the genera *Ruminococcus_1*, *Christensenellaceae_R-7_group*, *Ruminococcaceae_NK4A214_group*, *Alistipes*, *Bacteroides*, and *Akkermansia* were the most dominant microbial taxa in the gut microbial communities irrespective of breed type (Fig. 2). The relative abundances of the main phyla did not differ between both rabbit breeds, except for the phylum Verrucomicrobia, which showed a higher abundance in Elco rabbits (Additional file 2: Table S2). This could be explained by the presence of the phyla Firmicutes, Bacteroidetes, and Proteobacteria as they are significantly affected by diet factors (e.g., dietary fibre type and level) [20, 21] and our rabbits were reared under identical conditions, receiving the same diet. Similarly, Li et al. found that individuals of the same horse breed raised under different feeding regimes showed tremendous variations in the relative abundances of Firmicutes and Bacteroidetes but not in those of Verrucomicrobia [17]. Moreover, our previously study has found that the phylum Verrucomicrobia is related to nutrient extraction and intestinal health status of meat rabbits [22].

Likewise, the relative abundances of most dominant genera did not differ between the two rabbit breeds, but the greater abundances of *Akkermansia*, *Lachnospiraceae_ruminantium_group*, *Subdoligranulum*, and *Ruminococcus_2* were found in Elco rabbits (Additional file 2: Table S2). The genus *Akkermansia* belongs to the phylum Verrucomicrobia and its abundance has varied in different pig breeds from the same pig farm [23]. Importantly, *Akkermansia* could be involved in the formation of a protective mucosal layer that contributes to deal with inflammatory processes [24]. The relative abundance of *Subdoligranulum* and *Ruminococcus_2* has also been found to differ in different broiler chicken lines from the same poultry farm [25]. Additionally, *Ruminococcus* species have been reported to improve the immune response in rabbits [26].

At the OTU level, nineteen OTUs showed significantly different abundances between rabbit breeds, and over 65% of OTUs were annotated to the families Ruminococcaceae, Lachnospiraceae, and Bacteroidales_S24-7_group (Fig. 3). Org et al. demonstrated that genetic background of mice could explain a substantial amount of the variation of microorganism of these three families when mice were raised in a uniform environment [27]. In addition, Zeng et al. found that variations in the abundance of these three families were associated with body weight of rabbits [28].

At the species level, of the 23 species whose abundances differed significantly between Elco and Ira rabbits (Fig. 4), 16 belonged to the families Ruminococcaceae and Lachnospiraceae, which are known to ferment dietary fiber to produce short-chain fatty acids (SCFAs) [29, 30]. In this study, several well-known SCFAs-producing species were identified. For example, abundant *Coprococcus comes* inhabits in Ira rabbits, which produces acetate and exhibits multiple kinase activities (e.g., butyrate kinase) that play an important role in butyrate production [31]. *Ruminococcus faecis* is a butyrate-producing bacterium showing greater abundance in Ira rabbits, which has also been linked to acetate and propionate production [32]. Additionally, *Ruminococcus callidus* and *Lachnospiraceae bacterium NK4A136*, which are important butyrate producers, were more abundant in the gut of Elco rabbits [33, 34]. Due to SCFAs widely participate in physiological and pathophysiological interactions between the gut microbiota and the host [35], we hypothesized that these breed-associated SCFAs-producing species should play a central role in the health, development, and growth of rabbits, and could be considered as potential biomarkers for production performance improvement [36]. Indeed, previous studies have demonstrated that *Coprococcus*, *Ruminococcus*, and *Lachnospiraceae* species are intimately correlated with growth performance of meat rabbits [18, 37].

Differentially enriched functional features of the gut microbiome between Elco and Ira rabbits were also uncovered (Fig. 5). KOs related to bacterial chemotaxis, two-component system, and metabolic pathways of pentose phosphate, fructose, mannose, and branched chain amino acids (BCAAs, valine, leucine, and isoleucine) were more abundant in Elco rabbits, whereas KOs correlated with ABC transporters and metabolic pathways of galactose, glycerolipid, lysine, and aromatic amino acids (AAAs, phenylalanine, tyrosine, and tryptophan) were overrepresented in Ira rabbits. Bacterial chemotaxis and two-component system are essential for colonization and proliferation of gut microorganisms, and thus play important roles in energy cross-talk between gut microbiota and the host [25]. The pentose phosphate pathway is the major metabolic route that degrades non-digestible polysaccharides into oligosaccharides and monosaccharides fuels the central carbon metabolism [38]. A recent mouse gut microbiota study suggested that the presence of the pentose phosphate pathway was related to the host genetic background [39]. ABC transporters belong to transport system super family, which are widely distributed (from prokaryotes to eukaryotes) and evolutionarily conserved [40]. A similar study in sheep indicated that the abundance of microbial ABC transporters differed between Tibetan and Small Tail Han breeds grown under the same environmental conditions [41]. In addition, the metabolic pathways of carbohydrates (fructose, mannose, and galactose), amino acids (BCAAs, AAAs, and lysine), and lipids (glycerolipid) have been shown to differ among farm animal breeds grown under the standardized feeding conditions [6, 42–45]. The breed-associated KEGG pathways showed substantial overlap with the breed-associated KOs. However, several metabolic processes correlated with the host genetic background were found. For example, gene expression of the host cholesterol-7α-hydroxylase (CYP7A1), oxysterol-7α-hydroxylase (CYP7B1), and sterol-27-hydroxylase (CYP27A1) gene expressions can regulate the biosynthesis of gut microbial primary and secondary bile acid [46]. The metabolism of gut microbial vitamin B6 is modulated by different host genotypes [47].Additionally, the biosynthesis route of peptidoglycan provides essential substances that interact with host peptidoglycan recognition proteins (PGRPs), which play important roles in regulating metabolism and immune homeostasis [48]. These breed-associated differences in the functional profiles of the gut microbiome suggest the potential superiorities in manipulation of gut microbiome through selective breeding to promote the well-being and production performance of meat rabbits.

To identify breed-associated metabolites and further understand the underlying differences in basic metabolic processes in Elco and Ira rabbits, we investigated the characteristics of serum metabolomics (Additional file: Fig. S1, Fig. 6). We found that most of the metabolites that differed between rabbit breeds belonged to fatty acids, amino acids, and organic acids involved in distinct metabolic processes. Certain fatty acids are not only influenced by the host genetic background, but are also important biomarkers of production traits in animals. For instance, heptadecanoic and pentadecanoic acid are derived from fatty acid oxidation pathway, which differed between Jersey and Holstein breeds, and are associated with residual feed intake (RFI) status [49]. Serum nervonic acid, which is affected by the genotypes of dairy cow, is involved in fatty acid biosynthesis, and correlated with reproductive performance [50]. Specific amino acid concentrations in the biofluid and the corresponding metabolic pathways vary with different animal breeds, affecting the health and production performance of animals. For example, Liao et al. found that significant differences in levels of glutamine, cysteine, and glutamic acid among different beef cattle breeds led to alterations in the metabolism of cysteine, methionine, and glutamate, which are related to heat stress adaptability [8]. Wang et al. indicated that plasma alanine and proline were potential biomarkers for feed efficiency in Duroc and Landrace pigs due to their important roles in the metabolism of alanine, arginine, and proline [51]. Other studies have demonstrated that serum concentrations of glycine, histidine, lysine, and serine were associated with diverging RFI of different broiler chicken lines which may be attributed to their contributions to protein biosynthesis and ammonia recycling [52, 53]. Additionally, certain organic acids, such as 4-aminobutyric acid, 3-phenylpropanoic acid, and phenylacetic acid are involved in many biological processes: modulating glucose and lipid metabolism, regulating energy homeostasis, and exerting antioxidant, anti-inflammatory, and neuroprotective actions [54–56]. Thus, such breed-associated metabolites, which may be correlated with the health status and production traits of meat rabbits, could be considered as candidate biomarkers for breed improvement.

Previous studies have indicated that an altered serum metabolome profile could reflect differences in the gut microbiome of animals [11, 16, 57], which consistent with our findings (Fig. 7). In both rabbit breeds, ten dominant species had the largest number of significant correlations with metabolites, where seven species belonged to the families Lachnospiraceae (e.g., *Lachnospiraceae bacterium NK4A136* and *Lachnospiraceae bacterium CAG:215*) and Ruminococcaceae (e.g., *Ruminococcus sp. CAG:579* and *Ruminococcaceae bacterium mt9*). Likewise, 22 dominant metabolites had the largest number of significant associations with species, which were attributed to fatty acids (e.g., heptadecanoic acid, erucic acid, and linoleic acid), amino acids (e.g., L-lysine, L-cystine, and L-kynurenine), and organic acids (e.g., pristanic acid, 4-aminobutyric acid, and DL-3-hydroxybutyric acid). These results suggest that, in meat rabbits, host breeds can shape gut microbiome and serum metabolome, implying that interactions among host-gut microbiome- serum metabolome are important.

Our study has limitations as we analysed only two rabbit breeds, but it provides important evidence of the effect host breeds have on the gut microbiome and serum metabolome in meat rabbits and unravels a number of microbial species and serum metabolites that could be considered as candidate biomarkers for breed improvement in meat rabbits. In this context, further studies aiming to understand the underlying mechanisms of how host breeds modeling the gut microbiome and serum metabolome are needed. In addition, future studies aiming to validate such biomarkers in a large population with multiple rabbit breeds should also be considered.

## Conclusions

The current study provides information on the effect host breeds have on gut microbiome and serum metabolome of meat rabbits. Differences in gut microbial features (such as, diversity, microbial taxa, and functional capacities) and serum metabolites were detected between Elco and Ira rabbits. Even though such breed-associated differences constitute a small part of the complex biological processes, our study provides information that could aid future studies in determining the genetic basis of microbial and metabolome profiles variation, improving our knowledge of host-gut microbiome-serum metabolome interactions and how it influences the health and production traits of meat rabbits.

## Methods

### Experimental animals and sample collection

Fifteen rabbits (8 males and 7 females) with similar age (72 ± 2 days) were randomly selected from Elco and Ira breed in the rabbit farm of Wanjia Animal Husbandry Co., Ltd., Longyan, China. All rabbits were fed with the same commercial pellet diet (Additional file 2: Table S1) under the same raising environment conditions. All rabbits were healthy and had not received antibiotics, anticoccidial drugs, probiotics or prebiotics before hard fecal samples were collected. Three rabbits (2 males and 1 female) were randomly selected from each group for metagenomic sequencing and jugular vein blood collection. Blood samples were refrigerated on ice after collection for 1 h and serum was obtained by centrifugation at 2000 rpm for 10 min. All samples were snap frozen in liquid nitrogen for transportation and stored at − 80 °C until further utilization. At the end of the study, all rabbits (80 ± 2 days) were transported to the local slaughterhouse, stunned with electronarcosis and quickly bled by cutting the jugular veins and carotid arteries.

### 16S rRNA gene sequencing

Total genomic DNA was isolated from feces using the QIAamp Fast DNA Stool Mini Kit (QIAGEN, Germany) according to the manufacturer’s instructions. The quantity and quality of DNA was detected by using the Nanodrop ND-2000 spectrophotometer (Thermo Fisher Scientific, USA) and 1% agarose gel electrophoresis, respectively. The fusion primers 341F (5′-CCTACGGGNGGCWGCAG-3′) and 806R (5′- GGACTACHVGGGTATCTAAT-3′) were used to amplify the V3-V4 hypervariable region of the 16S rRNA gene under the annealing temperature of 55 °C with 28 cycles. The products of amplification were purified, and then sequenced on Hiseq-2500 platform (Illumina, USA) according to the manufacturer’s manuals. Quality control of raw data including filter out the primers, barcodes, and low quality sequences was accomplished by QIIME (v.1.9.1) [58]. High-quality paired-end reads (quality score ≥ 20) were assembled into tags by using FLASH (v.1.2.11) [59]. To avoid potential sequencing depth bias, the library size of microbial sequences of each sample was rarefied to 40,000 tags [60]. Tags with > 97% sequence identity were clustered into operational taxonomic units (OTUs) using USEARCH (v.10.0) [61]. Taxonomic category assignments of OTUs were performed by using SILVA database (v.132) [62]. The alpha and beta diversity indices were calculated using Mothur (v.1.41.1) and QIIME (v.1.9.1), respectively [58, 63].

### Metagenomic sequencing

According to the manufacturer’s instructions (Illumina, USA), a pair-end (PE) DNA library was constructed for each sample. Sequencing was performed on an Illumina Hiseq-4000 platform. Fastp (v.0.19.4) was used to quality control, adapter trimming, and low-quality reads filtering of raw reads [64]. High quality reads were assembled into contigs by using the MEGAHIT (v.1.1.3) [65]. Open reading frames (ORF) prediction was performed using the contigs with more than 200 bp in length by MetaGeneMark (v.2.10) [66]. Cd-hit (v.4.6.1) was used to exclude the redundant genes from the predicted ORFs to construct the non-redundant gene catalogue [67]. Gene abundance was calculated by mapping the high quality reads against the non-redundant gene catalogue using MOCAT (v2.0) [68]. Taxonomic category assignments of the genes were performed by aligning against non-redundant (NR) database using DIAMOND (v.0.9.24) [69]. KEGG Orthologies (KOs) and KEGG pathways annotation information from Kyoto Encyclopedia of Genes and Genomes (KEGG) database were obtained by GhostKOALA [70].

### Serum metabolomics profiling

Serum samples were used for untargeted metabolomics analysis by ultra-performance liquid chromatography system coupled with quadrupole time of flight mass spectrometry (UPLC-QTOFMS). Serum samples were preprocessed as the following modified protocol [71]. 100 μl of serum from each sample was precipitated by 300 μl methanol precooled to − 20 °C. After mixing with a vortex, all samples were incubated at − 20 °C for 30 min and centrifuged at 13,000 rpm for 20 min at 4 °C to obtain the supernatant. The supernatant (500 μl) was collected and freeze-dried for storage. The dried supernatants were resolved in 200 μl of 15% methanol (diluted by water) and transferred into the sampling vials for UPLC-QTOFMS (Waters, USA) analysis. Additionally, the quality control (QC) sample was created by mixing an aliquot of equal volume for each sample.

The Acquity UPLC system (Waters, USA) was used for chromatographic analysis. 2 μl prepared sample was injected into BEH C18 column (100 mm × 2.1 mm, 1.7 μm; Waters). To avoid the potential instability of the system and monitor analytical stability, QC sample was injected at the initial phase of analyzing. Under both positive and negative electrospray ion condition, the flow rate was 400 μl/min at column temperature 40 °C, all samples were eluted using a linear gradient from 100% solvent A (0.1% formic acid in water) to 100% solvent B (acetonitrile). After separation by UPLC, a Q-TOF Premier (Waters, USA) equipped with the electrospray ionization (ESI) source operating in positive and negative mode (Waters, USA) was used for mass spectrometry analysis. For both ionization modes, MS parameters were as follows: source temperature was set at 120 °C, desolvation temperature gas at 400 °C, capillary voltage at 2.5 kV. The scan range was from 50 to 1200 m/z with a scan time of 0.3 s. Leucine enkephalin (556.2771 m/z in ESI+, 554.2615 m/z in ESI-) was used as lock mass correction at a flow rate of 15 μL/min for each sample. System control and data acquisition was performed by using MassLynx (Waters, USA).

The raw data was processed by the Progenesis QI (Waters, USA) for peak alignment to obtain a peak list consist of the retention time, m/z, and peak area [72]. Based on retention time and the m/z data pairs, both ion intensity of each peak and a matrix consist of sample names, ion intensities, and arbitrarily assigned peak indices were obtained. The matrix was further trimmed by filtering out peaks with missing values in more than 75% of samples and those with isotope ions to obtain consistent variables. The qualified peaks were normalized to the QC sample by using support vector regression algorithm in R package MetNormalizer [73]. To assess the repeatability of metabolomic data sets, the coefficient of variation of metabolites in the QC samples was set at a threshold of 25%. Partial least squares discriminant analysis (PLS-DA), identification of significantly different metabolites (Wilcoxon test with FDR correction), and KEGG pathways enrichment analysis was performed by using MetaboAnalyst 4.0 web server [74].

### Statistical analysis

To identify the effect of host breeds, age and gender on gut microbial communities, redundancy analysis (RDA) was performed by using the vegan package in R. Wilcoxon rank sum test with false discovery rate (FDR) correction was performed to detect differences in microbial diversity indices and relative abundances of microbes at different taxonomic levels between Elco and Ira rabbits. The significant differences in relative abundances of microbial species and functional capacities between Elco and Ira rabbits were uncovered by using linear discriminant analysis effect size (LEfSe) analysis. To make heatmap more legibility to show the differential functional features, the relative abundances were transformed to enrichment scores as previously described by Contrepois et al. [75]. Spearman rank sum correlation analysis with FDR correction was used to calculate the correlation coefficients between breed-associated species and metabolites [76]. Excepted the differential OTUs were visualized using iTOL [77], the other plots were generated in R.

## Supplementary Information

**Additional file 1 **: **Fig. S1.** PLS-DA plot based on the serum metabolic profilings of Elco and Ira rabbits.

**Additional file 2 **: **Table S1.** Composition of the commercial pellet diet. **Table S2.** Differences in the relative abundances of dominant phyla and predominant genera. **Table S3.** OTUs showing significantly different abundances in Elco and Ira rabbits. **Table S4.** Differentially enriched functional capacities in Elco and Ira rabbits. **Table S5.** Significant differences in abundances of serum metabolites of Elco and Ira rabbits.

## Data Availability

We submitted the sequencing data to the SRA database in NCBI under with accession numbers: SRR13247207, SRR13247208, SRR13247209, SRR132472010, SRR132472011, and SRR132472012.

## Abbreviations

16S rRNA: 16S ribosomal ribonucleic acid
UPLC-QTOFMS: Ultra-performance liquid chromatography system coupled with quadrupole time of flight mass spectrometry
OTU: Operational taxonomic unit
RDA: Redundancy analysis
LEfSe: Linear discriminant analysis effect size
KEGG: Kyoto Encyclopedia of Genes and Genomes
KOs: KEGG Orthologies
FDR: false discovery rate
SCFAs: Short-chain fatty acids
QC: quality control
ESI: Electrospray ionization
PLS-DA: Partial least squares discriminant analysis
