# Supplementary figures and images for: Effect of host breeds on gut microbiome and serum metabolome in meat rabbits
Source: BMC Vet Res. 2021 Jan 7;17:24. doi: 10.1186/s12917-020-02732-6 (PMC7791989; doi:10.1186/s12917-020-02732-6)

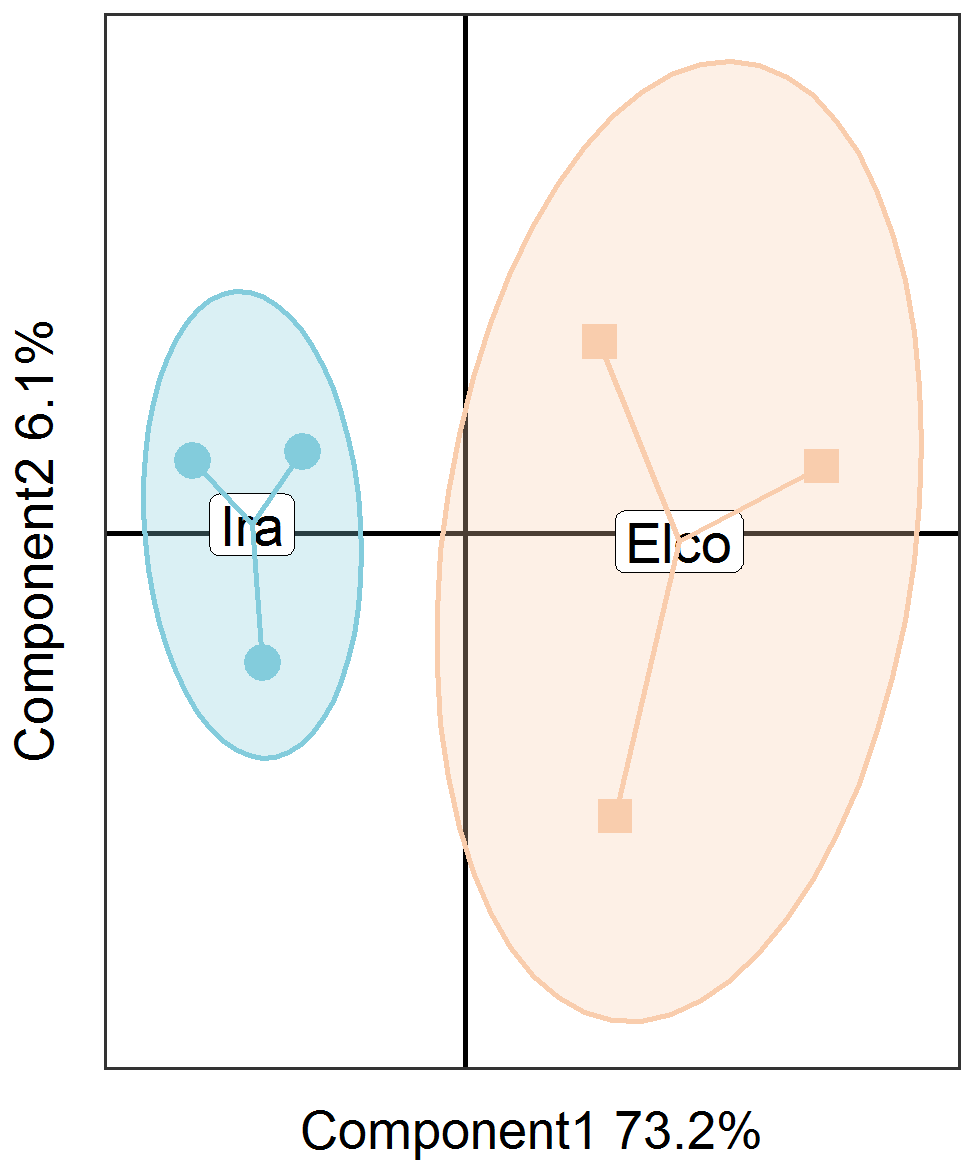

Supplement: Supplementary file 1 — Additional file 1 : Fig. S1. PLS-DA plot based on the serum metabolic profilings of Elco and Ira rabbits. [file 12917_2020_2732_MOESM1_ESM.tiff]
